# Adipose-derived stem cells extracellular vesicles enhance diabetic wound healing via CCN2/PI3K/AKT pathway: therapeutic potential and mechanistic insights

**DOI:** 10.1186/s13287-025-04354-x

**Published:** 2025-06-15

**Authors:** Yu-lu Zhou, Shingo Ogura, Hao Ma, Rong-bin Liang, Shao-yihan Fang, Yue-ming Wang, Yan Wo, Wen-jin Wang, De-Wu Liu

**Affiliations:** 1 Medical Center of Burn Plastic and Wound Repair, The First Affiliated Hospital, Jiangxi Medical College, Nanchang University, Nanchang, Jiangxi China; 2 Department of Plastic and Reconstructive Surgery, Shanghai Ninth People’s Hospital, Shanghai Jiao Tong University School of Medicine, Shanghai, China; 3 Department of Anatomy and Physiology, School of Medicine, Shanghai Jiao Tong University, Shanghai, China; 4 Department of Ophthalmology, Sir Run Run Shaw Hospital, Zhejiang University School of Medicine, Hangzhou, China

**Keywords:** Adipose-derived stem cells extracellular vesicles (ADSCs-EVs), Diabetic wound healing, Angiogenesis, Cellular communication network factor 2 (CCN2), PI3K/AKT signaling pathway

## Abstract

**Background:**

Adipose-derived stem cells extracellular vesicles (ADSCs-EVs) hold significant promise in tissue repair and regeneration. While they have been reported to enhance diabetic wound healing, the precise mechanisms remain unclear.

**Methods:**

ADSCs-EVs were isolated via ultracentrifugation and characterized through transmission electron microscopy, Western blot, and nanoparticle tracking analysis. Their effects on human umbilical vein endothelial cells (HUVECs) and RAW 264.7 macrophages were assessed in vitro, focusing on cell proliferation, migration, tube formation, and macrophage polarization. A diabetic rat wound model was used to evaluate their therapeutic impact on wound healing and angiogenesis, with histological and immunofluorescence analyses. mRNA sequencing identified Cellular communication network factor 2(CCN2) as a key upregulated gene, leading to further exploration of its role in ADSCs-EVs-mediated angiogenesis and wound healing via the PI3K/AKT pathway. Gene silencing (si-CCN2) and pharmacological inhibition (LY294002) were employed both in vitro and in vivo.

**Results:**

ADSCs-EVs were successfully isolated and characterized. In vitro, ADSCs-EVs promoted HUVEC proliferation, migration, and tube formation, and facilitated macrophage polarization to the M2 phenotype. In vivo studies using a diabetic rat wound model confirmed the pro-healing effects of ADSCs-EVs, including enhanced angiogenesis, granulation tissue formation, and accelerated wound closure. mRNA sequencing revealed that CCN2 expression was significantly upregulated in diabetic wound tissues treated with ADSCs-EVs. Further experiments showed that inhibiting CCN2 expression (si-CCN2) or blocking the PI3K/AKT pathway (LY294002) partially suppressed HUVEC proliferation, migration, tube formation, and angiogenesis, and counteracted the pro-healing effects of ADSCs-EVs.

**Conclusions:**

ADSCs-EVs promote diabetic wound healing through the CCN2/PI3K/AKT pathway, offering a promising therapeutic target for diabetic wound repair.

**Supplementary Information:**

The online version contains supplementary material available at 10.1186/s13287-025-04354-x.

## Introduction

Diabetes is a pervasive chronic metabolic disease affecting over 536 million individuals worldwide, with projections reaching 793 million by 2045 [1, 2]. Among its complications, diabetic foot ulcers (DFUs) pose a significant clinical challenge due to their multifactorial origins—combining peripheral neuropathy, vascular dysfunction, and infection—and are associated with high recurrence rates (over 40% within one year) as well as progression to amputation or mortality [3–5]. Despite advancements in therapeutic strategies such as debridement, growth factors, and surgical interventions, clinical outcomes remain suboptimal, highlighting the urgent need for innovative approaches to improve DFU healing [6, 7].

Impaired angiogenesis and immune dysfunction are central to diabetic wound pathophysiology, limiting oxygen supply, prolonging inflammation, and delaying healing [8]. Streptozotocin (STZ)-induced diabetic rat models are widely used to study these mechanisms, as they mimic key features of human diabetic wounds, providing a valuable platform to explore therapeutic strategies targeting vascular and macrophage dysfunction [9]. Among the key regulators of vascular and immune function during wound repair, Cellular Communication Network Factor 2 (CCN2, also known as Connective Tissue Growth Factor, CTGF) plays a pivotal role. It promotes angiogenesis, facilitates ECM deposition, and orchestrates the transition from inflammation to tissue regeneration, making it essential for normal wound healing [10, 11]. In diabetic wounds, reduced CCN2 expression is strongly associated with impaired angiogenesis and delayed repair [12, 13]. Emerging evidence links CCN2 to the PI3K/AKT signaling pathway, a key regulator of angiogenesis and vascular stability, particularly in the context of other diseases such as breast cancer [14]. The chronic inflammatory microenvironment in diabetic wounds is known to suppress PI3K/AKT activity, further impairing healing [15, 16]. While the involvement of CCN2 in reactivating the PI3K/AKT pathway in diabetic wounds remains unclear, its pro-angiogenic effects in other pathological conditions suggest it may represent a promising therapeutic target for restoring vascular function.

Recent advances in regenerative medicine have identified extracellular vehicles (EVs), particularly those derived from adipose-derived stem cells (ADSCs), as promising therapeutic agents for chronic wounds [17]. ADSCs-EVs exhibit potent pro-angiogenic and immunomodulatory effects and offer distinct advantages over cellular therapies, such as reduced immunogenicity and improved stability [18, 19]. To elucidate the underlying mechanisms of ADSCs-EVs in diabetic wound healing, our mRNA sequencing analysis of ADSCs-EVs-treated diabetic wound tissues revealed a significant upregulation of CCN2 expression.

In this study, we aim to validate the role of the CCN2/PI3K/AKT pathway in mediating the pro-healing effects of ADSCs-EVs on diabetic wounds. By combining mRNA sequencing with in vitro and in vivo experiments, we investigate the molecular mechanisms underlying ADSCs-EVs' therapeutic potential, focusing on their ability to promote angiogenesis and wound repair. This research is expected to provide critical insights into the regenerative mechanisms of ADSCs-EVs, laying the foundation for developing novel therapeutic strategies to improve clinical outcomes for patients with chronic diabetic wounds.

## Materials and methods

### Ethics approval and consent to participate

All procedures were approved by the Laboratory Animal Ethics Committee of the Ninth People's Hospital, Shanghai Jiao Tong University School of Medicine, under the project title "Adipose-Derived Stem Cells Extracellular Vesicles Enhance Diabetic Wound Healing in Rats" (Approval No. SH9H-2024-A9-1, approved on February 22, 2024). The work has been reported in line with the ARRIVE guidelines 2.0. [20]

The HUVECs (PCS-100–010) used in this study were purchased from ATCC, a globally recognized cell repository operating under an ISO 9001:2015-certified quality management system (Certificate Number: FM 516121, issued by BSI). This certification ensures that the acquisition, preservation, and distribution processes are conducted according to rigorous quality and ethical standards, providing confidence in the reliability of the materials. All experiments involving HUVECs in this study fully adhere to institutional and international ethical regulations. (For detailed information, see ATCC: https://www.atcc.org/).

### Isolation, culture, and identification of adipose stem cells (ADSCs)

Adipose-derived mesenchymal stem cells (ADSCs) were isolated from 6 to 8-week-old male Sprague–Dawley (SD) rats’ groin subcutaneous fat using sterile methods. Cultured in vitro to the third passage, their adipogenic and osteogenic potentials were assessed by Oil Red O and Alizarin Red staining. Surface markers CD29, CD44, CD45, and CD90 were analyzed via flow cytometry (CUBE8, Sysmex, Japan) to confirm their stem cell phenotype.

### Isolation and identification of extracellular vesicles from adipose stem cells (ADSCs-EVs)

Adipose-derived mesenchymal stem cells (ADSCs) at passages three to five (P3–P5) were cultured in serum-free DMEM for 48 h. The supernatant was collected, and extracellular vesicles (ADSC-EVs) were extracted via ultrafiltration [21, 22]. Specifically, the supernatant was processed using a 100 kDa ultrafiltration tube at 3500 g/min and maintained at 4 °C. This ultrafiltration step was repeated multiple times, with the retained sample being rinsed and resuspended in PBS after each cycle, until the filtrate in the ultrafiltration tube became colorless. To further improve purity, the retained solution was passed through a 0.22 μm filter, ensuring the removal of residual impurities or non-vesicular particles. The final ADSC-EV preparation was resuspended in PBS and stored at − 80 °C for further use. ADSC-EVs morphology was analyzed using Transmission electron microscopy (TEM), particle size via nanoparticle tracking analysis (NTA), and markers CD9, CD63, CD81 and TSG101 confirmed by Western blotting. Protein concentration was quantified using a bicinchoninic acid (BCA) protein assay kit.

### Cell uptake in vitro

To evaluate the intracellular uptake of ADSCs-EVs, the cell membrane dye CM-Dil (BALB, Beijing, China) was used to label the EVs, which were then resuspended in complete culture medium. HUVECs (ATCC, PCS-100–010) and RAW264.7 murine macrophages (ATCC, TIB-71) were purchased from the American Type Culture Collection (ATCC, USA) and seeded in confocal culture dishes and incubated for 24 h in complete culture medium containing the resuspended EVs. After incubation, cells were washed with PBS to remove extracellular EVs and then fixed with 4% paraformaldehyde. Nuclei were stained with 6-diamidino-2-phenylindole (DAPI, 40728ES03, Yeasen Biotech, China) for 10 min, and the cytoskeleton was stained with TRITC-phalloidin (40734ES75, Yeasen Biotech, China) for 20 min. The intracellular uptake of EVs was observed using a confocal laser scanning microscope (FV3000, Olympus, Japan).

### Cell proliferation assay

The effects of ADSCs-derived extracellular vesicles (ADSCs-EVs) on cell proliferation under normal (NG) and high-glucose (HG) conditions were assessed using the Cell Counting Kit-8 (CCK-8; Beyotime, China) assay. In the first set of experiments, HUVECs and RAW264.7 cells were seeded at 5 × 10^3^ cells per well in 96-well plates and maintained under NG (5.5 mM glucose) or HG (30 mM glucose) conditions [23]. Cells were divided into three groups: NG control, HG control, and HG + ADSCs-EVs (50 μg/mL). After 24, 48, 72 h of treatment, 10 μL CCK-8 reagent was added to each well, and the absorbance was measured at 450 nm following a 1–2-h incubation.

To further investigate the underlying mechanisms, additional treatment groups were introduced in a second set of experiments: HG + ADSCs-EVs + si-CCN2 and HG + ADSCs-EVs + LY294002 (10 μM). For siRNA transfection, cells were pretreated with si-CCN2 (50 nM) or its negative control (si-NC) using Lipofectamine 3000 for 24 h prior to the addition of ADSCs-EVs. Absorbance at 450 nm was measured as described above. All experiments were conducted in triplicate, with three independent biological replicates performed for each group.

### Scratch assay

HUVECs migration was evaluated using a scratch wound healing method. The experimental setup, cell group assignments, ADSCs-EVs treatment, and si-CCN2/LY294002 interventions were consistent with the CCK-8 experiment described above. Briefly, HUVECs were cultured in 6-well plates until 90–100% confluence, after which a scratch was made across the monolayer using a 200 μL pipette tip. Detached cells were removed by washing twice with PBS, and fresh media containing the respective treatments was added. Migration rates were assessed at 0-, 12-, 24-, and 48-h using ImageJ, (National Institutes of Health, USA) and relative migration was calculated as [(initial distance − remaining distance)/initial distance] × 100%. All experiments were performed independently in triplicate.

### Tubulogenesis assay

Tubulogenesis assays were performed in Matrigel-coated 96-well plates to evaluate capillary-like tube formation. HUVECs (2 × 10^4^ cells/well) were seeded and treated using the same concentrations and conditions as described in the cell proliferation assay. Images were captured at 6–12 h for analysis of tube formation, including branch nodes and total length, using ImageJ with the Angiogenesis Analyzer plugin.

### Diabetic wound model establishment

Male Sprague Dawley rats (6–8 weeks, 200 ± 10 g) were maintained under standard conditions (22 ± 2 °C, 55 ± 5% humidity, and 12-h light/dark cycle) with food and water provided ad libitum. Diabetes mellitus (DM) was induced by a single intraperitoneal injection of freshly prepared streptozotocin (50 mg/kg, S0130, Sigma-Aldrich) diluted in citrate buffer (pH 4.5) after a 12-h fast [9]. For the non-diabetic control (NC) group, rats were injected with citrate buffer alone as a control. Individual rats were considered the experimental unit in this study.

Blood glucose levels were measured on days 7 and 14 post-injection using a glucometer (OneTouch, China), and rats with blood glucose levels ≥ 16.7 mmol/L were used as diabetic models. Two full-thickness dorsal wounds (2.5 cm in diameter) were created under sodium pentobarbital anesthesia (35 mg/kg) using a sterile biopsy punch, reaching the panniculus carnosus layer. Silicone fixation rings were sutured around the wounds to control contraction [24].

### In Vivo interventions

Rats were divided into two phases to determine the efficacy of ADSC-derived extracellular vesicles (ADSCs-EVs) and explore their mechanisms in diabetic wound healing. In Phase 1, rats were assigned to the NC + PBS (non-diabetic control, n = 6), DM + PBS (diabetic control, n = 6), or DM + ADSCs-EVs (0.5 mg/ml, 10 µL per injection site, n = 6) groups. In Phase 2, rats in the DM + ADSCs-EVs group were further divided into DM + ADSCs-EVs + si-CCN2 (50 nM siRNA, n = 6) and DM + ADSCs-EVs + LY294002 (10 µM PI3K inhibitor, n = 6) groups. All drugs and treatments were delivered through local injection targeting the wound edge at two evenly distributed points. ADSCs-EVs were administered as a single injection (0.5 mg/ml on Day 0) to evaluate their sustained functional effects. For each wound, a total of 20 µL was locally injected, with 10 µL delivered at each of two points around the wound margin to ensure adequate tissue exposure. CCN2 silencing was achieved by delivering 50 nM siRNA with Entranster^™^-in vivo (18668–11-1, Engreen, China) [25] via two separate injections (Day 0 and Day 7). Similarly, LY294002 (10 µM in PBS with 0.1% DMSO, prepared fresh for each injection) was administered following the same two-injection regimen (Day 0 and Day 7) to maintain its inhibitory effects throughout the critical phases of wound healing. Rats were humanely euthanized by intraperitoneal injection of sodium pentobarbital at a dose of 100 mg/kg, following the guidelines of the American Veterinary Medical Association (AVMA). Death was confirmed by the cessation of vital signs, including respiration and cardiac activity. Euthanasia was performed on Days 7 and 14 of the experiment, and wound tissues were subsequently collected for histological, immunohistochemical, and molecular analyses.

### Quantitative real-time PCR (qRT-PCR)

HUVECs were cultured in 6-well plates under standard conditions and treated to divide into three groups: HG control, HG + ADSCs-EVs (50 μg/mL) and HG + ADSCs-EVs + si-CCN2 (50 nM). Total RNA was extracted from cells using the TRIzol reagent (Invitrogen, USA) according to the manufacturer’s protocol. The quality and concentration of RNA were assessed using a NanoDrop spectrophotometer (Thermo Fisher Scientific, USA), and only samples with an A260/A280 ratio between 1.8 and 2.0 were used for subsequent experiments. For cDNA synthesis, 1 μg of total RNA was reverse transcribed into complementary DNA (cDNA) using the PrimeScript RT reagent kit (Takara, Japan) following the manufacturer’s instructions. The reverse transcription reaction was carried out at 37 °C for 15 min, followed by enzyme inactivation at 85 °C for 5 s. qRT-PCR was conducted using SYBR Green PCR Master Mix (Bio-Rad, USA) on the CFX96 Real-Time PCR Detection System (Bio-Rad, USA). The primer sequences used for the Homo species are listed below: CCN2 forward: 5′-GACCUGGAAGAGAACAUUATT-3′, CCN2 reverse: 5′- UAAUGUUCUCUUCCAGGUCTT -3′; and GAPDH forward: 5′- TGACTTCAACAGCGACACCCA-3′, GAPDH reverse: 5′- CACCCTGTTGCTGTAGCCAAA-3′. Relative gene expression levels were normalized to GAPDH as the internal control and calculated using the 2^-ΔΔCt method.

### Western blot analysis

Proteins from rat skin tissue samples were extracted using RIPA lysis buffer (Beyotime, Shanghai, China), and protein concentrations were determined with a BCA Protein Assay Kit (Beyotime Biotechnology, China). Equal amounts of total protein (20 μg per lane) from four experimental groups (DM + PBS, DM + ADSCs-EVs, DM + ADSCs-EVs + si-CCN2, and DM + ADSCs-EVs + LY294002) were separated via 10% SDS-PAGE and transferred onto PVDF membranes (Millipore, IPVH00010, Germany). For each target protein, samples from all groups were loaded on the same gel to ensure comparability. Membranes were blocked with 5% non-fat milk in TBST for 1 h at room temperature, followed by overnight incubation at 4 °C with primary antibodies against CCN2, PI3K, p-PI3K, AKT, p-AKT, VEGF-A, and β-actin (Proteintech, Wuhan, China). After TBST washes, membranes were incubated with HRP-conjugated secondary antibody (1:3000, RGAM001, Proteintech, Wuhan, China) for 1 h at room temperature. Protein bands were visualized using the Clarity ECL Substrate (Bio-Rad, USA), and membranes were exposed to X-ray films (Kodak, USA) in a darkroom. Exposure times ranged between 10 s and 1 min, optimized based on signal strength. Films were developed using an automatic processor and analyzed for visible protein bands. Developed films were dried, labeled, and archived for long-term storage. Relative protein expression was quantified using ImageJ software by normalizing the signal intensity of each target protein band to that of β-actin, which was used as the reference protein. Relative protein expression was quantified by normalizing target protein signals to actin using ImageJ software.

### Wound photography and histological staining

Wound healing progression was photographically recorded on Days 0, 3, 7, 10, and 14 post-operation, and wound closure rates were quantified using ImageJ software. Skin tissue samples were collected on Days 7 and 14, fixed in 4% paraformaldehyde (PFA, Beyotime, Shanghai, China) for 24 h, embedded in paraffin, and sectioned. Hematoxylin and eosin (H&E, G1003) and Masson’s trichrome staining (G1006) reagents were purchased from Servicebio (Wuhan, China) and applied to the paraffin sections. Stained sections were observed using high-resolution microscopy (OLYMPUS DP70, NIKON, Japan), and measurements were performed using Slide Viewer software (3DHISTECH Ltd, Hungary).

### Immunofluorescence staining

To evaluate angiogenesis during wound healing, tissues from the wound site and adjacent normal skin were collected on Days 7 and 14 post-wounding, fixed in 4% paraformaldehyde (PFA, Beyotime, Shanghai, China) at room temperature, and rinsed with PBS. Immunofluorescence staining was performed using an anti-CD31 antibody (Abcam) to label vascular endothelial cells, with nuclei counterstained by 4',6-diamidino-2-phenylindole (DAPI, Sigma-Aldrich, MO, USA). Images were acquired using a Leica SP5 confocal microscope (Mannheim, Germany).

### Enzyme-linked immunosorbent assays (Elisa)

To evaluate angiogenesis and inflammatory cytokine expression during diabetic wound healing, inflammatory cytokines (IL-6, IL-10, and TNF-α) and VEGF-A levels were measured on Day 7,14 post-wounding using specific ELISA kits (Enzyme-linked Biotechnology Co., Shanghai, China). Homogenized wound tissues and standards were prepared and added to antibody-coated microwell plates according to the manufacturer’s instructions. Optical density (OD) at 450 nm was detected using an ELISA reader, and the concentrations of VEGF-A, IL-6, IL-10, and TNF-α were calculated based on their respective standard curves.

### Transcriptomic sequencing of rat skin tissue

Skin tissues were collected from diabetic wound model rats following 7-day interventions with PBS (n = 6) or ADSCs-EVs (n = 6), snap-frozen in liquid nitrogen, and stored at − 80 °C. Total RNA was extracted using Trizol^®^ Reagent (Qiagen, Germany), and RNA quality was assessed with a Bioanalyzer 5300 (Agilent) and NanoDrop ND-2000. Samples with OD260/280 ratios of 1.8–2.2, OD260/230 ≥ 2.0, RQN ≥ 6.5, and 28S:18S ≥ 1.0 were selected for further analysis. High-quality RNA was used for mRNA sequencing following purification and library preparation by Majorbio (Shanghai, China). cDNA synthesis was performed with SuperScript (Invitrogen), and mRNA-seq libraries were constructed and sequenced using the NovaSeq X Plus platform (Illumina) with a paired-end 150 bp read length.

A total of 12 RNA samples (PBS group, n = 6; ADSCs-EVs group, n = 6) were sequenced. After sequencing and initial quality control, four samples per group were selected for downstream statistical analysis based on RNA quality, sequencing depth, and data homogeneity to ensure reliable and representative results. Differentially expressed genes (DEGs) were identified using DESeq2, with thresholds of log2 (Fold Change)|≥ 1 and a false discovery rate (FDR) < 0.05. Gene Ontology (GO) and Kyoto Encyclopedia of Genes and Genomes (KEGG) pathway enrichment analyses were conducted using Goatools and the Python Scipy package. Principal component analysis (PCA) was performed with unit variance scaling in Python to reduce dimensionality and visualize sample clustering.

### Statistical analysis

The data analysis was conducted by the same researchers responsible for conducting the experiment, and group identities were known during the analysis. Although the experiment was not blinded, all data were recorded objectively using predefined measurement protocols to minimize subjective bias. All experiments were conducted in triplicate (n ≥ 3 biological replicates per group) to ensure robust statistical validity and reproducibility. Statistical analyses were performed using GraphPad Prism 8.0 (GraphPad Software LLC, San Diego, USA). The normality of data distributions was assessed with the Shapiro–Wilk test. For comparisons between two groups, an unpaired t-test was used for normally distributed data, while the Wilcoxon rank-sum test was applied for non-normal data. For datasets involving three or more groups, one-way analysis of variance (ANOVA) was utilized. Post-hoc comparisons following ANOVA were conducted using the Bonferroni correction. All results are represented as mean ± standard deviation (Mean ± SD) and statistical significance was established at p < 0.05(* p < 0.05;**p < 0.01;***p < 0.001).

## Results

### Isolation and identification of ADSCs and ADSCs-EVs

**Fig. 1:**
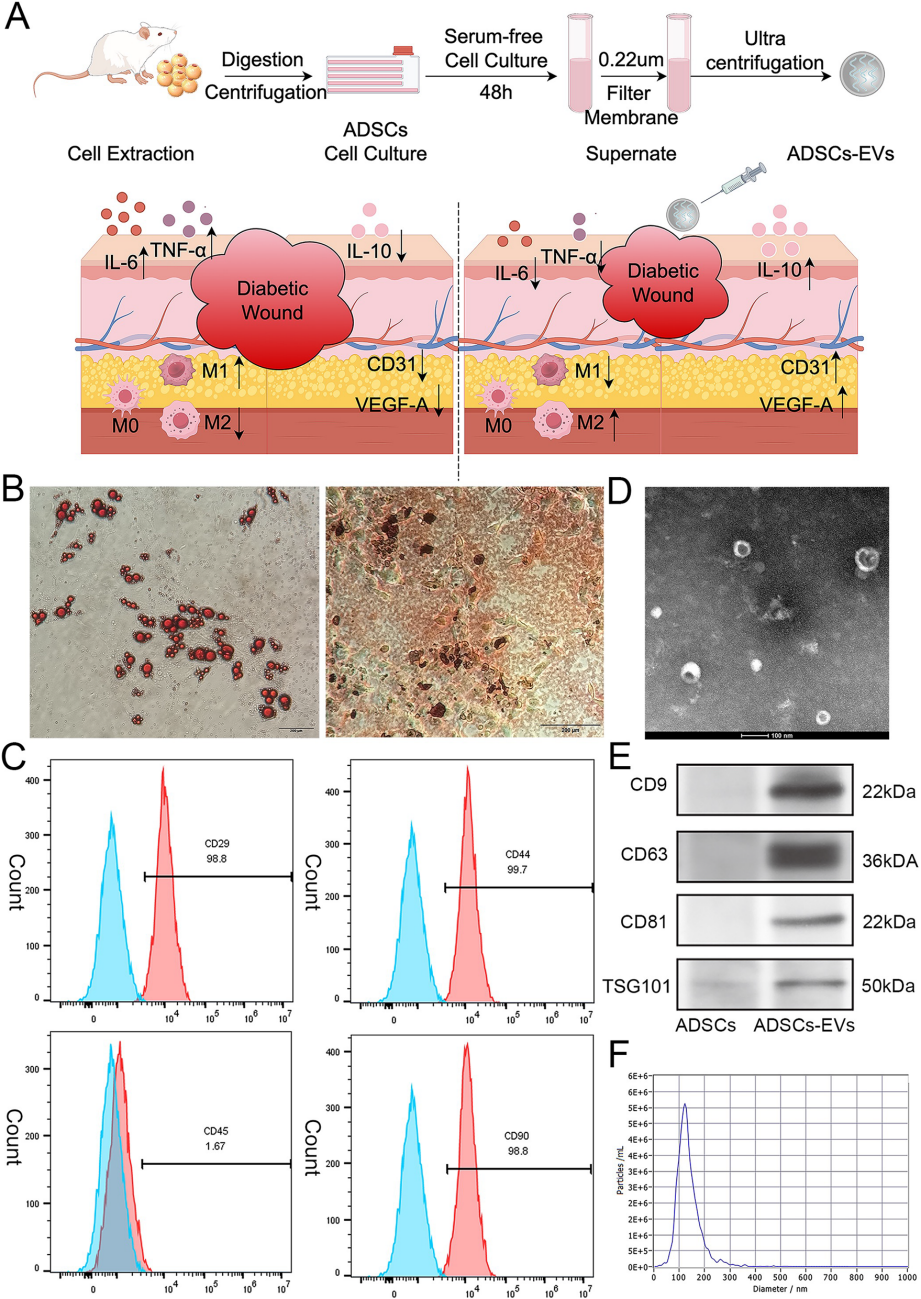
Characterization of ADSCs and ADSCs-EVs. **A** Schematic representation of macrophage polarization dysfunction, angiogenesis impairment, and the pro-healing effects of ADSCs-EVs. **B** Differentiation of ADSCs into adipocytes and osteoblasts, confirmed by Oil Red O and Alizarin Red S staining. **C** Flow cytometry showing ADSCs expressed CD29, CD44, CD90, and lacked CD45. **D** TEM images of ADSCs-EVs showing their spherical bilayer structure. **E** Western blot detecting ADSCs-EVs markers (CD9, CD63, CD81, TSG101). **F** NTA analysis of ADSCs-EVs indicating diameters of 30–200 nm

ADSCs and ADSCs-EVs were successfully extracted as shown in the schematic diagram (Fig. 1A), which also provides an overview of the study’s central focus. The diagram illustrates macrophage polarization dysfunction and impaired angiogenesis in diabetic wounds, as well as the pro-healing effects of ADSCs-EVs intervention. ADSCs were further confirmed by their ability to differentiate into adipocytes and osteoblasts, validated by Oil Red O and Alizarin Red S staining (Fig. 1B). Flow cytometry analysis demonstrated that ADSCs expressed the mesenchymal stem cell markers CD29, CD44, and CD90, while lacking the hematopoietic marker CD45 (Fig. 1C).

The extracellular vesicles derived from ADSCs (ADSCs-EVs) were examined using transmission electron microscopy (TEM), which showed their characteristic spherical bilayer membrane structure with heterogeneous size distribution (Fig. 1D). Western blot analysis confirmed the presence of typical EVs surface markers, including CD9, CD63, CD81, and TSG101 (Fig. 1E). Further characterization by Nanoparticle Tracking Analysis (NTA) revealed that the diameters of ADSCs-EVs ranged between 30 and 200 nm (Fig. 1F).

### In Vitro effects of ADSCs-EVs on high glucose-treated HUVECs and RAW 264.7

**Fig. 2:**
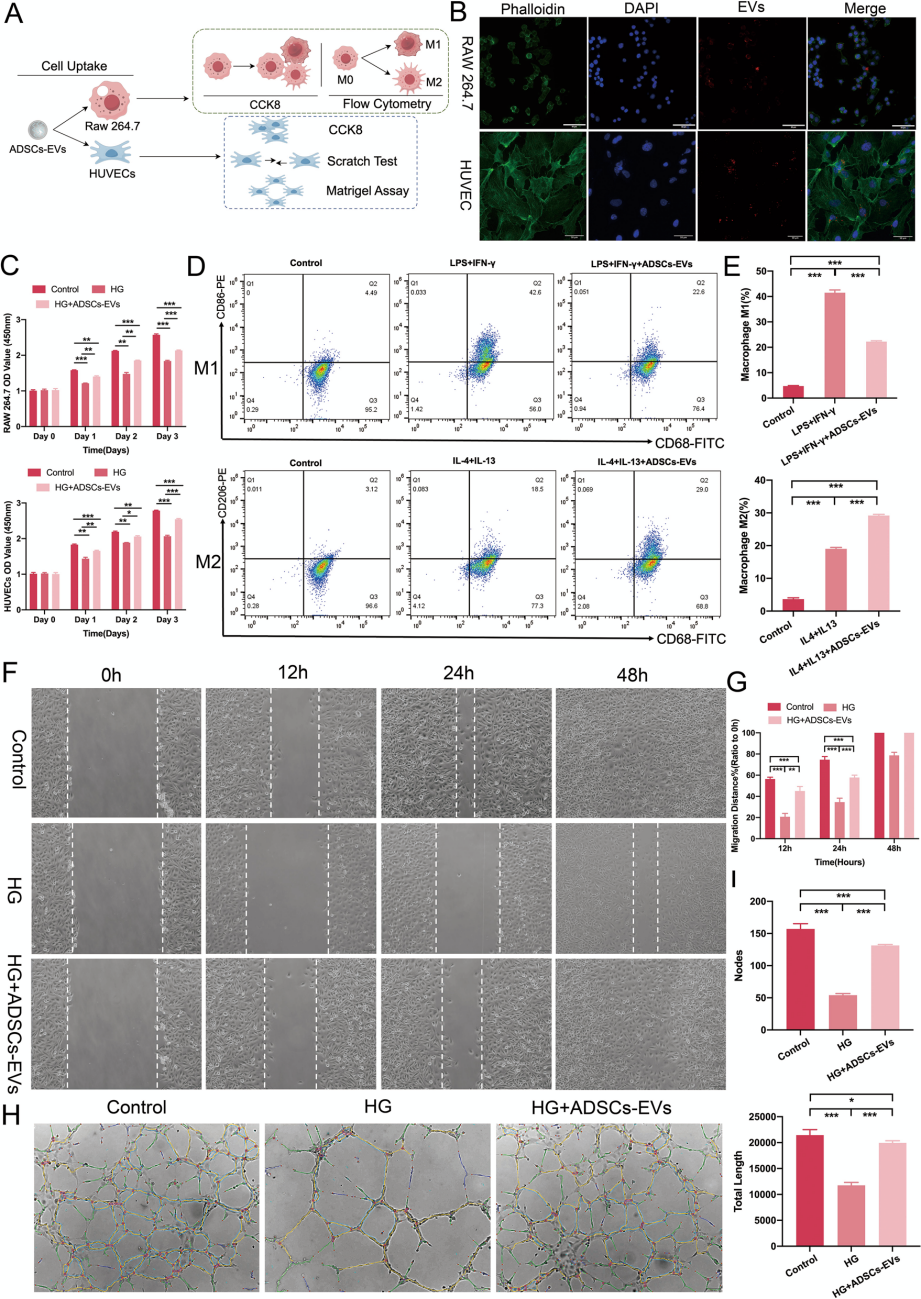
ADSCs-EVs counteract high-glucose-induced dysfunction in HUVECs and RAW264.7 macrophages. **A** Experimental schematic illustrating the investigation of ADSCs-EVs effects on proliferation, polarization, migration, and angiogenic functions under high-glucose conditions. **B** Confocal microscopy showing the uptake of DIL-labeled ADSCs-EVs by HUVECs and RAW264.7 macrophages after 12 h of co-incubation. **C** CCK-8 proliferation assay demonstrating the enhanced proliferation of HUVECs and RAW264.7 cells following ADSCs-EVs treatment compared to the high-glucose (HG) control group. **D**, **E** ADSCs-EVs modulated macrophage polarization, reducing M1 macrophages and increasing M2 macrophages, as shown by flow cytometry plots and quantification. **F**, **G** Scratch assay showing enhanced migration of HUVECs treated with ADSCs-EVs under high-glucose conditions, evidenced by increased wound closure rates at 12, 24, and 48 h. **H**, **I** Matrigel-based tube formation assays demonstrating improved angiogenesis in HUVECs following ADSCs-EVs treatment, with increased branch node numbers and total tube lengths compared to the HG control group. Statistical significance is represented as (*p < 0.05; **p < 0.01; ***p < 0.001)

Cell uptake: ADSCs-derived extracellular vesicles (ADSCs-EVs) were evaluated for their effects on high-glucose-treated Human umbilical vein endothelial cells (HUVECs) and RAW 264.7, a murine macrophage cell line, focusing on uptake, proliferation, polarization, migration, and angiogenesis (Fig. 2A). Confocal microscopy confirmed efficient uptake of DIL-labeled ADSCs-EVs by both cell types after 12 h of co-incubation, with vesicles localized around the nucleus (Fig. 2B).

Cell proliferation assay: CCK-8 assays demonstrated that ADSCs-EVs significantly enhanced proliferation under high-glucose (HG) conditions in both RAW 264.7 cells and HUVECs. Proliferation in RAW 264.7 cells increased significantly on day 1 and day 2 (p < 0.01) and was even more pronounced on day 3 (p < 0.001), compared to that in the HG control group (Fig. 2C). Similarly, HUVEC proliferation was restored, reversing HG-induced suppression, with significant effects observed on day 3 (p < 0.001).

Macrophage polarization: Flow cytometry analysis indicated that ADSCs-EVs effectively modulated macrophage polarization by significantly decreasing M1 macrophages (p < 0.001) and increasing M2 macrophages (p < 0.001, Fig. 2D, E).

HUVEC migration and angiogenesis: ADSCs-EVs markedly improved HUVEC migration, as shown by significantly enhanced wound closure rates in scratch assays (p < 0.001, Fig. 2F, G). Additionally, ADSCs-EVs robustly promoted angiogenesis, with a significant increase in tube branch points and total tube length in Matrigel assays (p < 0.001, Fig. 2H, I).

### In Vivo effects of ADSCs-EVs on diabetic wound healing

**Fig. 3:**
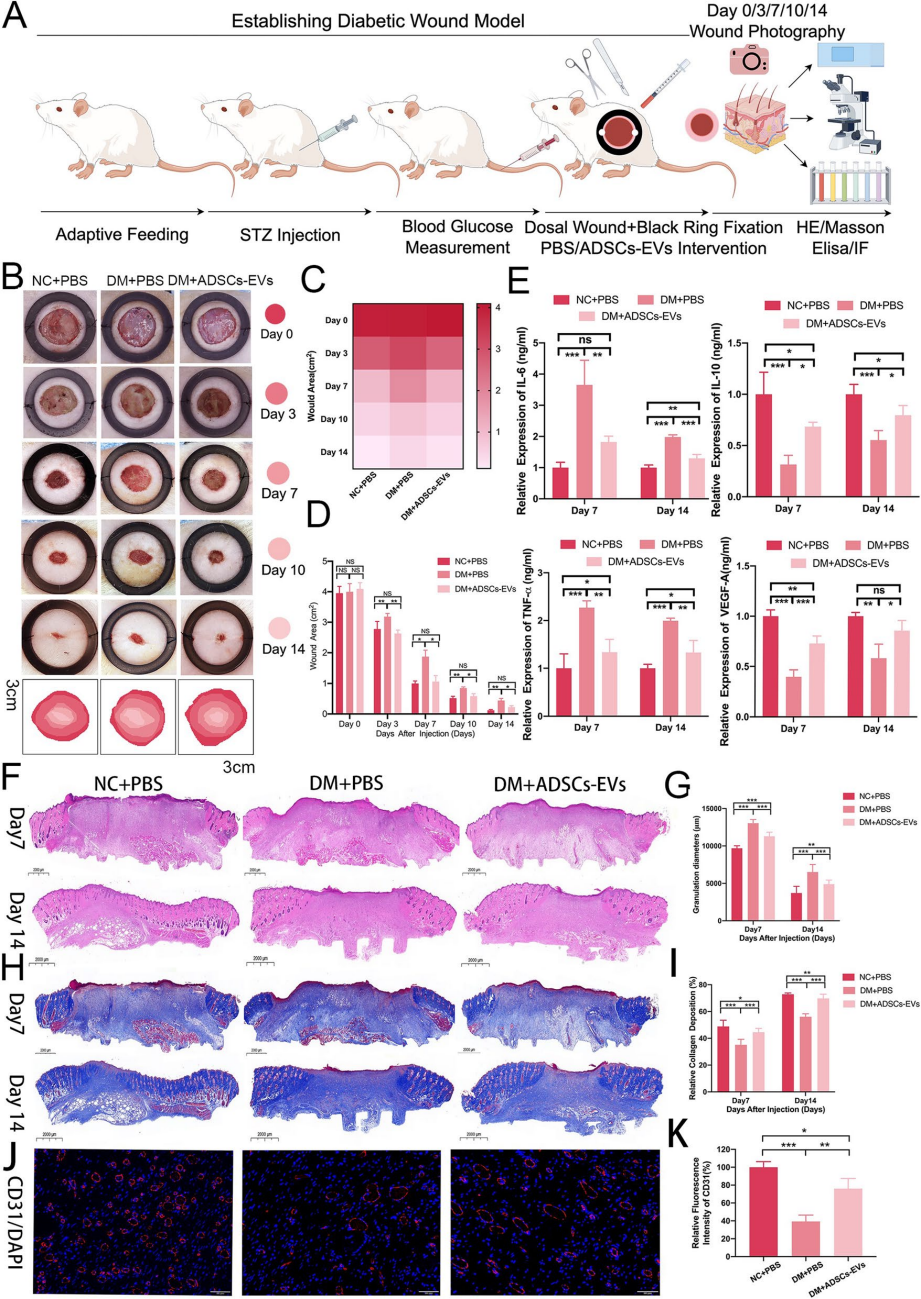
In Vivo Effects of ADSCs-EVs on Diabetic Wound Healing. **A** Schematic diagram depicting the diabetic wound model and experimental workflow for ADSCs-EVs treatment. **B** Representative photographs and corresponding fitting curves of wound healing at days 0, 3, 7, 10, and 14, showing accelerated wound closure in the DM + ADSCs-EVs group compared to the DM + PBS control group. **C**, **D** Heatmap visualizations and quantitative analyses of unhealed wound area reveal significant improvement in wound healing in the DM + ADSCs-EVs group starting from day 3 and continuing through day 14. **E** ELISA analysis of wound tissues demonstrates reduced pro-inflammatory cytokines (IL-6, TNF-α) and increased anti-inflammatory cytokine (IL-10), as well as elevated VEGF-A levels, in the DM + ADSCs-EVs group compared to the DM + PBS group. (F–H) H&E staining showing enhanced re-epithelialization and granulation tissue formation in the DM + ADSCs-EVs group at days 7 and 14. (**G**–**I** Masson’s trichrome staining demonstrating increased collagen deposition in wounds treated with ADSCs-EVs. **J**, **K** Immunofluorescence staining showing elevated CD31 expression in the DM + ADSCs-EVs group, indicating improved angiogenesis compared to the DM + PBS group. Statistical significance is represented as(*p < 0.05; **p < 0.01; ***p < 0.001)

A diabetic wound model was established to evaluate the therapeutic effects of ADSCs-EVs on wound healing (Fig. 3A). Representative photographs and fitting curves (Fig. 3B) demonstrated significantly accelerated wound healing in the DM + ADSCs-EVs group compared to controls. Heatmaps and statistical analysis of unhealed wound areas (Fig. 3C, D) showed a noticeable reduction in the ADSCs-EVs group from day 3 onwards (p < 0.01) and by day 14 (p < 0.05) wound areas were markedly smaller compared to controls.

ELISA analysis (Fig. 3E) revealed that ADSCs-EVs modulated inflammation by reducing pro-inflammatory cytokines (IL-6, TNF-α), while increasing anti-inflammatory IL-10 levels, and elevated VEGF-A levels, indicating enhanced angiogenesis (p < 0.05). Histological evaluations (Fig. 3F–I) showed improved re-epithelialization, granulation tissue formation, and collagen deposition in the ADSCs-EVs group, particularly at days 7 and 14 (p < 0.001). Furthermore, immunofluorescence staining (Fig. 3J, K) demonstrated significantly higher CD31 expression in the ADSCs-EVs group (p < 0.01), supporting enhanced angiogenesis and tissue repair.

### Transcriptomic sequencing of diabetic rat skin tissue

**Fig. 4:**
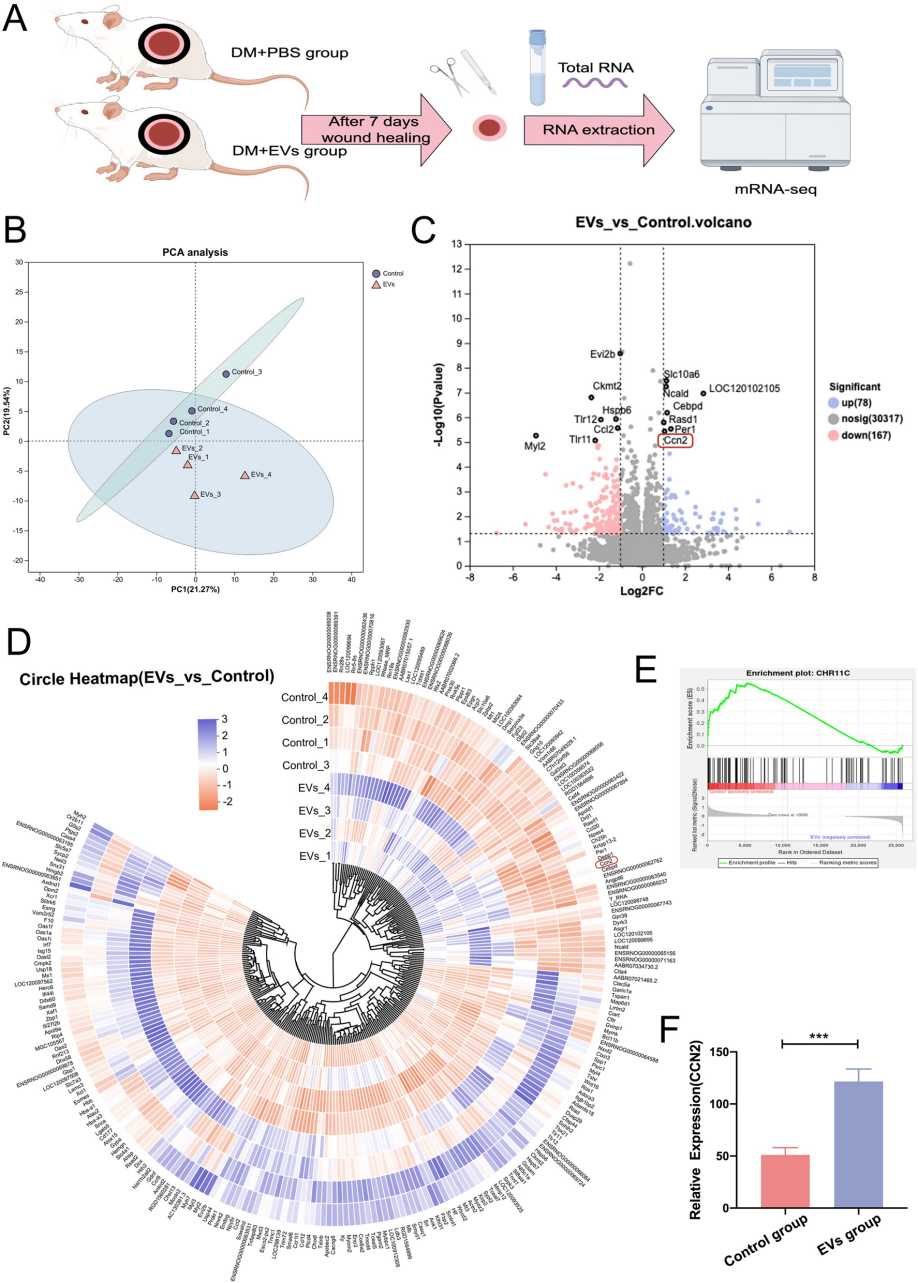
Transcriptomic analysis of diabetic wound tissues post ADSCs-EVs intervention. **A** Schematic workflow of mRNA sequencing on diabetic wounds 7 days after PBS or ADSCs-EVs treatment. **B** PCA analysis showing distinct transcriptomic profiles between DM + PBS and DM + ADSCs-EVs groups. **C** Differential expression analysis identified 245 DEGs, including 78 upregulated and 167 downregulated genes in the ADSCs-EVs group. A volcano plot highlights the top 14 DEGs with 7 upregulated and 7 downregulated genes. **D** Heatmap illustrating significantly upregulated CCN2 expression in the ADSCs-EVs group. **E** GSEA highlighting significant differences in gene expression pathways. **F** Statistical analysis confirmed higher Ccn2 expression in the DM + ADSCs-EVs group compared to DM + PBS controls, implicating CCN2 in ADSCs-EVs-mediated wound healing. Statistical significance is represented as (*p < 0.05; **p < 0.01; ***p < 0.001)

On day 7 post-intervention, mRNA sequencing revealed significant transcriptomic changes in diabetic wound tissues (Fig. 4A). PCA revealed distinct separation between the DM + PBS and DM + ADSCs-EVs groups, reflecting divergent transcriptomic profiles (Fig. 4B). Differential expression analysis revealed 245 DEGs, with 78 upregulated and 167 downregulated in the ADSCs-EVs group. To prioritize candidate genes, all DEGs were ranked based on their statistical significance (-log10 p-value). The top 20 genes were selected for further analysis to balance statistical rigor with biological relevance. Among these, 14 genes exhibited significant expression changes, with 7 upregulated and 7 downregulated. Functional screening of these genes, focusing on their known or potential roles in wound healing, revealed CCN2 (connective tissue growth factor) as a highly relevant target due to its critical involvement in processes such as angiogenesis, extracellular matrix (ECM) remodeling, and cell migration. CCN2 was also strongly upregulated in the ADSCs-EVs group, as demonstrated by the volcano plot and circular cluster heatmap (Fig. 4C, D). Gene Set Enrichment Analysis (GSEA) identified significant pathway-level differences (Fig. 4E). Ccn2 expression was significantly elevated in the ADSCs-EVs group compared to controls (p < 0.001, Fig. 4F). These findings suggest that ADSCs-EVs promote diabetic wound healing by modulating CCN2 expression.

### Role of the CCN2/PI3K/AKT signaling axis in the In Vitro effects of ADSCs-EVs on high glucose-treated HUVECs

**Fig. 5:**
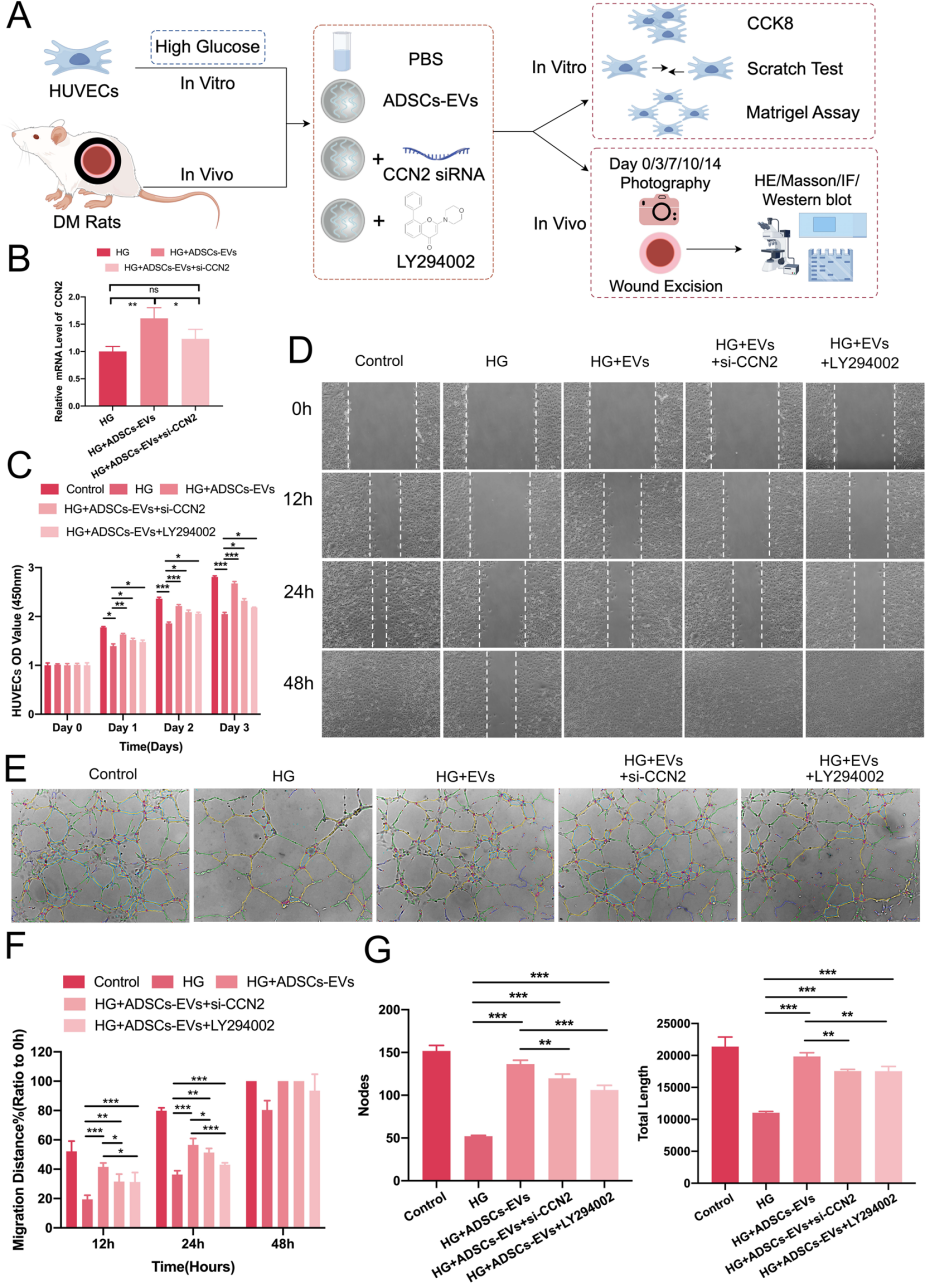
The CCN2/PI3K/AKT pathway is involved in the effects of ADSCs-EVs on high glucose (HG)-treated HUVECs. **A** Schematic of the experimental setup using si-CCN2 and the PI3K inhibitor LY294002 to validate pathway involvement. **B** qRT-PCR showing CCN2 upregulation by ADSCs-EVs and effective knockdown by si-CCN2 in HG conditions. **C** ADSCs-EVs significantly enhanced HUVEC proliferation under HG conditions, while this effect was reduced by si-CCN2 and LY294002. **D**, **F** In the scratch assay, ADSCs-EVs promoted HUVEC migration, whereas si-CCN2 and LY294002 inhibited this effect. Incomplete wound closure was observed in the HG + PBS and HG + ADSCs-EVs + LY294002 groups at 48 h. **E**, **G** The tube formation assay showed that ADSCs-EVs enhanced angiogenesis, increasing branch nodes and total tube length, effects that were partially reversed by si-CCN2 and LY294002. Statistical significance is represented as (*p < 0.05; **p < 0.01; ***p < 0.001)

Based on bioinformatics analysis (Fig. 4), ADSCs-EVs were shown to upregulate CCN2, a key regulator interacting with the PI3K/AKT signaling pathway. To validate the involvement of this axis in the effects of ADSCs-EVs on HUVECs and diabetic wound healing, functional experiments were performed using si-CCN2 and the PI3K inhibitor LY294002 (Fig. 5A). To validate the knockdown efficiency of si-CCN2, qRT-PCR analysis was performed in three groups: HG, HG + ADSCs-EVs, and HG + ADSCs-EVs + si-CCN2. The results showed that CCN2 mRNA expression was significantly upregulated in the HG + ADSCs-EVs group compared to the HG group (p < 0.001). Transfection with si-CCN2 effectively reduced CCN2 expression in the HG + ADSCs-EVs + si-CCN2 group (p < 0.001), confirming the efficiency of gene silencing and laying a solid foundation for subsequent functional assays (Fig. 5B).

In the CCK-8 assay, ADSCs-EVs significantly enhanced HUVEC proliferation under high glucose conditions (p < 0.0001, Fig. 5C), whereas both si-CCN2 and LY294002 partially suppressed this effect, indicating the involvement of the CCN2/PI3K/AKT axis (p < 0.05). Similarly, in the scratch assay, si-CCN2 and LY294002 significantly inhibited the pro-migratory effects of ADSCs-EVs, with a notable reduction in migration rates at 12 h and 24 h (p < 0.05), resulting in incomplete wound closure by 48 h (Fig. 5D, F). In the tube formation assay, si-CCN2 and LY294002 markedly reduced the pro-angiogenic effects of ADSCs-EVs under high glucose conditions. Compared to the ADSCs-EVs group, si-CCN2 and LY294002 treatments resulted in significant decreases in branch node numbers and total tube length (p < 0.001, Fig. 5E, G). These findings demonstrate that the enhanced proliferation, migration, and angiogenesis induced by ADSCs-EVs are mediated, at least in part, through the CCN2/PI3K/AKT signaling pathway, supporting their potential therapeutic role in diabetic wound healing.

### Role of the CCN2/PI3K/AKT signaling axis in the In Vivo effects of ADSCs-EVs on diabetic wound healing

**Fig. 6:**
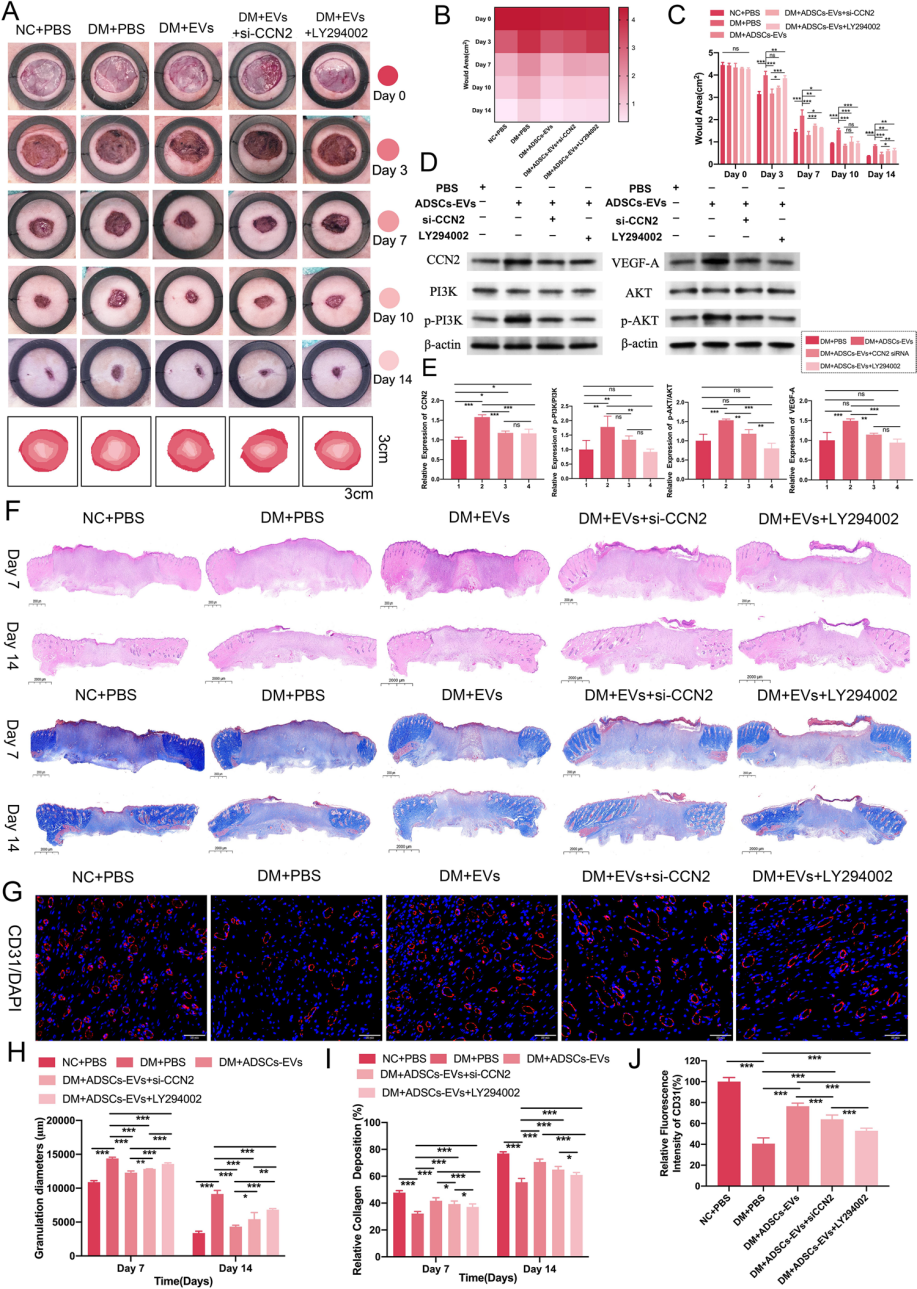
ADSCs-EVs accelerate diabetic wound healing via the CCN2/PI3K/AKT signaling pathway. **A** Representative wound images on days 0, 3, 7, 10, and 14 post-treatments, showing faster healing in the DM + ADSCs-EVs group compared to DM + PBS. Delayed healing was observed in DM + ADSCs-EVs + si-CCN2 and DM + ADSCs-EVs + LY294002 groups compared to DM + ADSCs-EVs but with better outcomes than DM + PBS. **B**, **C** Heatmap and statistical analysis of unhealed wound areas support these trends. **D** Western blot was performed to detect CCN2, VEGF-A, phosphorylated PI3K (p-PI3K), phosphorylated AKT (p-AKT), and their corresponding total proteins (PI3K, AKT). β-Actin was used as the internal loading control. Representative images from three independent experiments using the same batch of samples are shown. Full-length uncropped blots, including molecular weight markers and β-Actin, are available in Supplementary Information (Figure S1). **E** Quantification of target protein expressions normalized to β-actin. **F**, **H**, **I** H&E and Masson’s staining show enhanced granulation tissue formation and collagen deposition in DM + ADSCs-EVs compared to DM + PBS. These changes were reduced in DM + ADSCs-EVs + si-CCN2 and DM + ADSCs-EVs + LY294002 but remained higher than in DM + PBS. **G**, **J** CD31 staining demonstrates increased angiogenesis in DM + ADSCs-EVs, which was partially suppressed in si-CCN2 and LY294002 groups. Statistical significance is represented as (*p < 0.05; **p < 0.01; ***p < 0.001)

Representative photographs (Fig. 6A) showed that ADSCs-EVs significantly accelerated diabetic wound healing compared to controls, with the DM + ADSCs-EVs + si-CCN2 and DM + ADSCs-EVs + LY294002 groups exhibiting delayed but still improved healing relative to DM + PBS. Heatmap visualizations and statistical analyses (Fig. 6B, C) confirmed that ADSCs-EVs markedly reduced wound areas at all time points (p < 0.001). However, slower healing was observed in the DM + ADSCs-EVs + si-CCN2 and DM + ADSCs-EVs + LY294002 groups at days 3, 7, and 14 (p < 0.05–0.001), but differences were not significant at day 10 (p > 0.05).

Western blot analysis (Fig. 6D, E) revealed that ADSCs-EVs significantly upregulated CCN2 and VEGF-A expression and activated the PI3K/AKT pathway, with higher levels of CCN2, VEGF-A, p-PI3K/PI3K, and p-AKT/AKT ratios in the DM + ADSCs-EVs group compared to DM + PBS (p < 0.01–0.001). In contrast, si-CCN2 and LY294002 treatments attenuated these effects, showing reduced expression of CCN2, VEGF-A, and pathway activation markers compared to the DM + ADSCs-EVs group (p < 0.001), though still higher than in DM + PBS (p < 0.05–0.001).

Histological analyses (Fig. 6F) demonstrated that ADSCs-EVs promoted granulation tissue formation and collagen deposition, as shown by H&E and Masson’s trichrome staining (p < 0.001). While si-CCN2 and LY294002 groups also improved these parameters compared to DM + PBS (p < 0.001), the effects were diminished relative to DM + ADSCs-EVs. CD31 immunofluorescence staining (Fig. 6G, J) revealed enhanced angiogenesis in the DM + ADSCs-EVs group, with significantly higher vessel density compared to DM + PBS (p < 0.001). This pro-angiogenic effect was partially suppressed in the si-CCN2 and LY294002 groups (p < 0.001). These results collectively demonstrate that ADSCs-EVs promote diabetic wound healing by enhancing angiogenesis, granulation tissue formation, and collagen deposition via activation of the CCN2/PI3K/AKT signaling axis.

## Discussion

This study demonstrates that extracellular vehicles (EVs) derived from adipose-derived stem cells (ADSCs) enhance diabetic wound healing by creating a pro-repair microenvironment and promoting angiogenesis. Mechanistically, mRNA sequencing revealed that ADSCs-EVs treatment upregulated CCN2 expression. Functional validation, including in vitro (high-glucose-impaired HUVECs) and in vivo (diabetic wound rat models) experiments, showed that the pro-angiogenic and reparative effects of EVs were partially reversed by si-CCN2 or the PI3K inhibitor LY294002. Importantly, consistent inhibitory trends were observed in both systems, highlighting a functional link between CCN2 upregulation and PI3K/AKT pathway activation in EVs-mediated repair. These findings provide novel insights into the mechanisms of EVs in chronic wound healing and establish the CCN2/PI3K/AKT axis as a key target, highlighting the potential of ADSCs-EVs as a promising therapeutic strategy for refractory wounds with significant clinical implications.

Chronic wounds, especially diabetic wounds, pose substantial challenges for tissue repair due to impaired angiogenesis, insufficient resolution of inflammation, and defective extracellular matrix remodeling. Among the critical mediators of wound healing, CCN2 (Cellular Communication Network factor 2) plays a central role in orchestrating the transition from inflammation to proliferation by actively promoting angiogenesis and extracellular matrix deposition [10, 26]. In normal wound healing, CCN2 is prominently expressed in granulation tissue during the transition from the inflammatory phase to the proliferation phase, a process tightly regulated by TGF-β signaling and macrophage polarization toward the pro-repair M2 phenotype [27, 28]. However, diabetic wounds are dominated by persistent M1 macrophages, excessive neutrophil infiltration, and high TNF-α levels, which suppress TGF-β signaling and downregulate CCN2, delaying healing [29]. Given the central role of CCN2 in wound healing processes, restoring its expression represents a promising therapeutic strategy for addressing chronic wound healing deficits.

In this study, we demonstrated that extracellular vehicles (EVs) treatment effectively restores a pro-repair microenvironment in diabetic wounds, primarily through the upregulation of CCN2. EVs promoted macrophage polarization toward the M2 phenotype, elevated anti-inflammatory cytokine IL-10 levels, and suppressed pro-inflammatory cytokines IL-6 and TNF-α, collectively alleviating inflammation and facilitating tissue repair. Transcriptomic analysis revealed that EVs treatment reversed the downregulation of CCN2 in diabetic wounds, which was further corroborated by in vivo experiments. Specifically, diabetic wound rat models treated with EVs showed improved angiogenesis and accelerated wound healing, while silencing CCN2 using siRNA attenuated these effects, confirming its central role in the repair process. Consistently, in vitro experiments demonstrated that EVs significantly enhanced key endothelial cell functions, including proliferation, migration, and tube formation, all of which are indispensable for angiogenesis. Furthermore, studies employing synthetic CCN2 protein in diabetic wound healing provided additional validation of CCN2 as a critical mediator of tissue repair [30]. Interestingly, however, previous studies in systemic sclerosis (SSc) revealed that EVs downregulated CCN2 and TGF-β expression, alleviating excessive fibrosis and preventing ECM over-accumulation. This contrast indicates that EVs exert adaptive, disease-specific regulatory effects, enhancing CCN2 expression to promote angiogenesis in diabetic wounds, while suppressing it in SSc to prevent fibrotic progression [31].

ADSCs-EVs treatment was associated with significant enrichment of the PI3K/AKT signaling pathway in diabetic wounds, a key pathway controlling angiogenesis and cellular repair processes [16, 32]. Although CCN2 was not directly identified among the PI3K/AKT-related factors enriched in transcriptomic analysis, previous studies have demonstrated that CCN2 can function as an upstream activator of PI3K/AKT signaling in various cellular contexts, crucial for promoting endothelial cell migration, proliferation, and other biological processes [14]. For instance, Nelumbo nucifera leaf extract (NLE) suppresses angiogenesis by downregulating CCN2, thereby inhibiting the PI3K/AKT/ERK pathway and reducing VEGF and MMP2 expression [33]. In chondrocytes, CTGF enhances gap junction intercellular communication by upregulating connexin43 (Cx43) through PI3K/AKT activation, while pathway inhibition disrupts this process [34]. These findings highlight CCN2's essential role in modulating PI3K/AKT signaling to regulate angiogenesis, migration, and cell communication. To further substantiate the involvement of this pathway, we used the PI3K/AKT-specific inhibitor LY294002 in both in vitro and in vivo models. LY294002 partly abolished EVs-induced angiogenesis by significantly reducing HUVECs proliferation, migration, and tube formation in vitro. Similarly, in diabetic rat wounds, LY294002 partly inhibited CD31 expression, a marker of angiogenesis. These inhibitory effects aligned across both models, strongly suggesting that EVs-mediated repair relies, at least partially, on PI3K/AKT activation downstream of CCN2 upregulation.

This study demonstrates that extracellular vehicles (EVs) promote diabetic wound healing by linking CCN2 to the PI3K/AKT signaling pathway, a critical regulator of angiogenesis and tissue repair processes. Although the precise molecular regulators of this pathway remain unclear, our findings establish CCN2 as a potential therapeutic target and underscore the pivotal role of EVs in mediating repair. Beyond PI3K/AKT, previous studies suggest that CCN2 may interact with other critical signaling pathways, such as TGF-β [35] /Smad [36], Wnt/β-catenin [37], and VEGF/VEGFR [38], which regulate inflammation resolution, fibroblast activation, extracellular matrix remodeling, and angiogenesis. While these interactions were not directly investigated here, further studies could elucidate how CCN2 integrates these networks to optimize EVs-mediated tissue repair. Moreover, utilizing advanced molecular techniques such as gene editing or synthetic biology could further enhance the therapeutic potential of EVs. For instance, preconditioning adipose-derived stem cells (ADSCs) with recombinant human CCN2 (rhCCN2) or genetically engineering ADSCs may potentiate the pro-regenerative properties of their secreted EVs. Investigating how such interventions influence the molecular cargo and functional properties of ADSCs-EVs could help uncover innovative strategies to improve diabetic wound healing. Together, these future efforts will provide deeper mechanistic insights and help develop optimized, targeted EVs-based therapies for chronic wounds.

As our understanding of CCN2's role in diabetic wound healing grows, the therapeutic use of ADSCs-EVs hinges on addressing key challenges, including standardized dosage, EV cargo, and clinical translation [39]. In this study, 0.5 mg/mL ADSCs-EVs were injected locally (20 µL total) based on pilot experiments confirming efficacy and safety. Similar localized strategies have shown promise in enhancing bioavailability and wound healing [40, 41]. Future studies should explore dose optimization, timing, and repeated administration to better translate preclinical findings into clinical applications. In addition to dosage optimization, understanding the EV cargo composition represents another critical challenge. Beyond CCN2, ADSCs-EVs carry bioactive molecules, including miRNAs (e.g., miR-21 [42], miR-17–92 [43]) and lncRNAs (e.g., MALAT1 [44]), which synergistically enhance angiogenesis, tissue repair, and wound healing. Furthermore, the clinical translation of ADSCs-EVs is constrained by variability in EV cargo, standardization of isolation methods, and delivery strategies. Cargo heterogeneity [45] arises from donor-specific factors and differing isolation techniques, underscoring the need for standardized protocols to ensure reproducibility and regulatory compliance [46]. Delivery methods remain a challenge, but innovations like hydrogel scaffolds and engineered EVs offer potential solutions to enhance localization and therapeutic efficiency [47]. Addressing these hurdles is critical for bridging the gap between preclinical findings and reliable clinical applications, ultimately advancing ADSCs-EVs-based therapies for diabetic wound healing.

## Conclusions

This study demonstrates that ADSCs-EVs significantly enhance angiogenesis, a key process underlying diabetic wound healing. Mechanistically, we identify CCN2 as a critical mediator of these effects, functioning through the activation of the PI3K/AKT signaling pathway. These findings highlight ADSCs-EVs and the CCN2/PI3K/AKT axis as promising therapeutic targets for diabetic wound repair, providing an insight for future clinical translations.

## Supplementary Information

Additional file 1.

## Data Availability

The sequencing data generated during this study have been deposited in the NCBI Sequence Read Archive (SRA) under BioProject ID [PRJNA1209624] and are publicly accessible. All other datasets used and/or analyzed during the current study are available from the corresponding author upon reasonable request.

## Abbreviations

CCN2: Cellular communication network factor 2
ADSCs-EVs/EVs: Adipose-derived stem cell extracellular vesicles
HUVECs: Human umbilical vein endothelial cells
PI3K: Phosphatidylinositol 3-kinase
AKT: Protein kinase B(PKB)
VEGF: Vascular endothelial growth factor
HG: High glucose
NG: Nature glucose
DM: Diabetic mellitus
NTA: Nanoparticle tracking analysis
TEM: Transmission electron microscopy
