# Correction: Adipose-derived stem cells extracellular vesicles enhance diabetic wound healing via CCN2/PI3K/AKT pathway: therapeutic potential and mechanistic insights

**DOI:** 10.1186/s13287-025-04562-5

**Published:** 2025-08-06

**Authors:** Yu-lu Zhou, Shingo Ogura, Hao Ma, Rong-bin Liang, Shao-yihan Fang, Yue-ming Wang, Yan Wo, Wen-jin Wang, De-Wu Liu

**Affiliations:** 1 Medical Center of Burn Plastic and Wound Repair, The First Affiliated Hospital, Jiangxi Medical College, Nanchang University, Nanchang, Jiangxi China; 2 Department of Plastic and Reconstructive Surgery, Shanghai Ninth People’s Hospital, Shanghai Jiao Tong University School of Medicine, Shanghai, China; 3 Department of Anatomy and Physiology, School of Medicine, Shanghai Jiao Tong University, Shanghai, China; 4 Department of Ophthalmology, Sir Run Run Shaw Hospital, Zhejiang University School of Medicine, Hangzhou, China

**Stem Cell Research & Therapy (2025) 16:304**

10.1186/s13287-025-04354-x

The authors noticed that an incorrect version of Fig. 5D was inadvertently included in the original article.

To ensure full accuracy, the authors have carefully reviewed all original experimental images for this group, re-analyzed the data, and replaced both the representative image (Fig. 5D) and the corresponding statistical analysis (Fig. 5F).

**Fig. 5:**
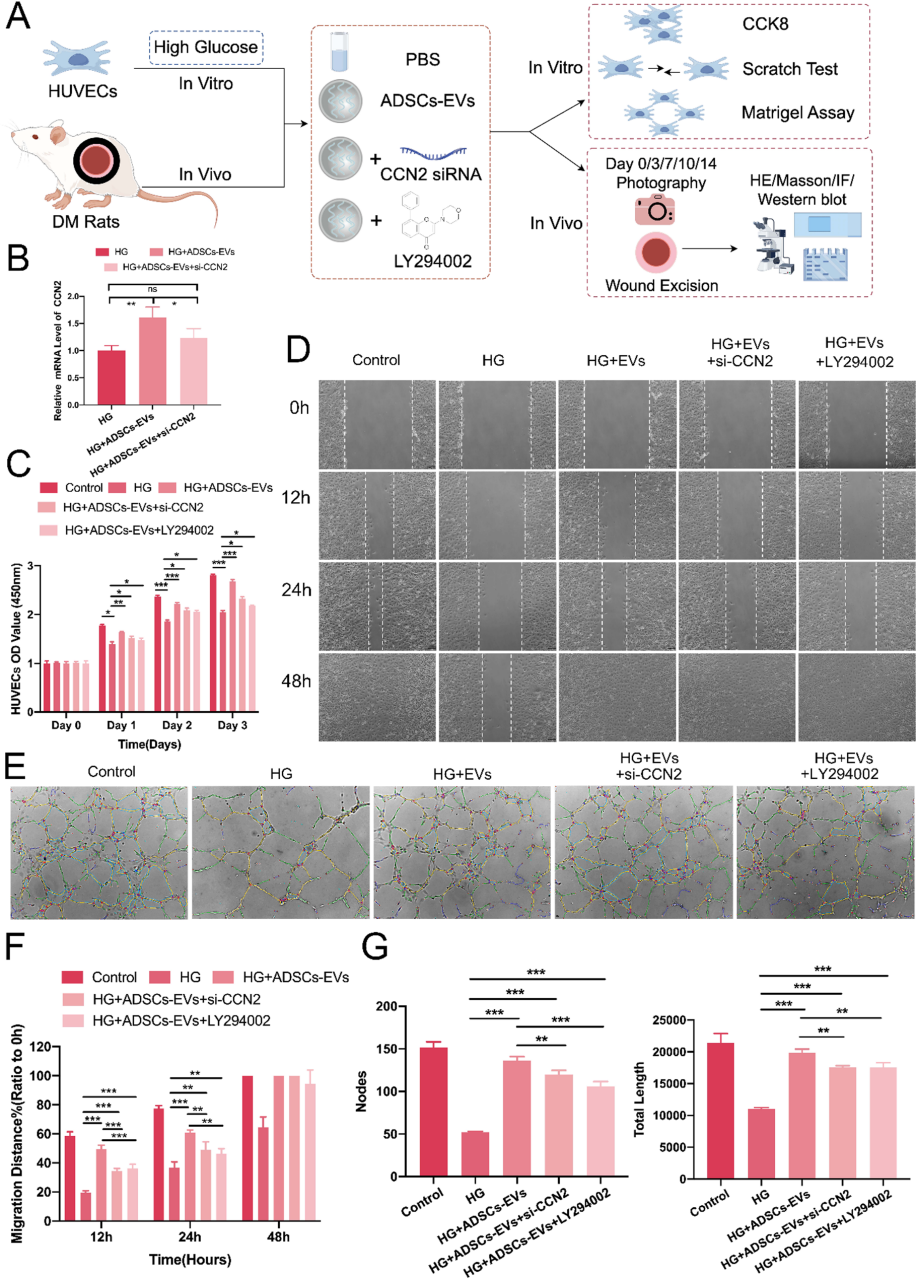
The CCN2/PI3K/AKT pathway is involved in the effects of ADSCs-EVs on high glucose (HG)-treated HUVECs. **A** Schematic of the experimental setup using si-CCN2 and the PI3K inhibitor LY294002 to validate pathway involvement. **B** qRT-PCR showing CCN2 upregulation by ADSCs-EVs and effective knockdown by si-CCN2 in HG conditions. **C** ADSCs-EVs significantly enhanced HUVEC proliferation under HG conditions, while this effect was reduced by si-CCN2 and LY294002. **D**, **F** In the scratch assay, ADSCs-EVs promoted HUVEC migration, whereas si-CCN2 and LY294002 inhibited this effect. Incomplete wound closure was observed in the HG + PBS and HG + ADSCs-EVs + LY294002 groups at 48 h. **E**, **G** The tube formation assay showed that ADSCs-EVs enhanced angiogenesis, increasing branch nodes and total tube length, effects that were partially reversed by si-CCN2 and LY294002. Statistical significance is represented as (*p < 0.05; **p < 0.01; ***p < 0.001)

The authors would like to emphasize that this correction does not affect the main conclusions of the article.

