# Supplementary figures and images for: Adipose-derived stem cells extracellular vesicles enhance diabetic wound healing via CCN2/PI3K/AKT pathway: therapeutic potential and mechanistic insights
Source: Stem Cell Res Ther. 2025 Jun 15;16:304. doi: 10.1186/s13287-025-04354-x (PMC12168405; doi:10.1186/s13287-025-04354-x)

Repricate 1

|            |   |   |   |   |
|------------|---|---|---|---|
| PBS        | + | — | — | — |
| ADSCs-EVs  | — | + | + | + |
| CCN2 siRNA | — | — | + | — |
| LY294002   | — | — | — | + |

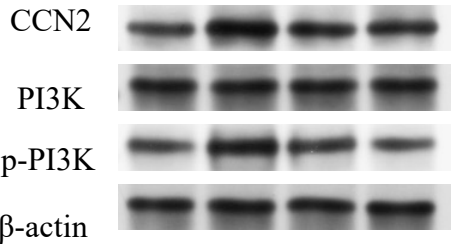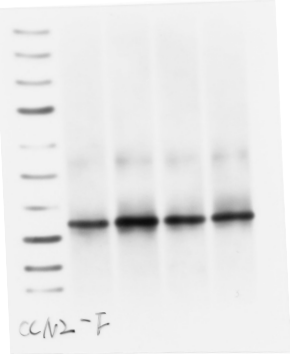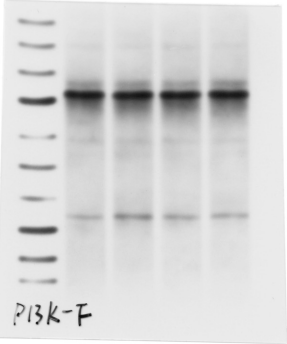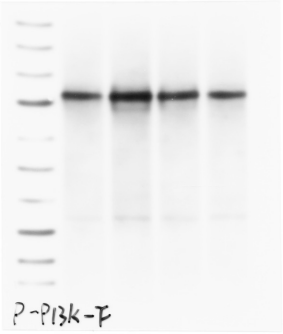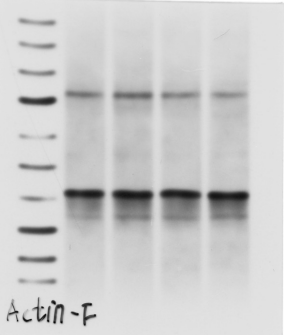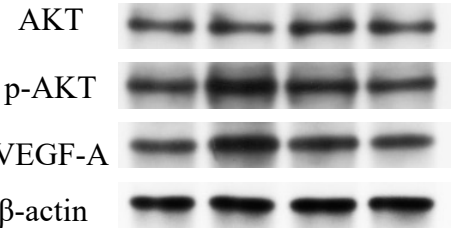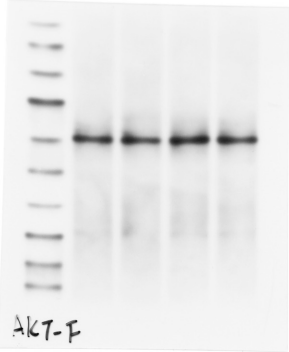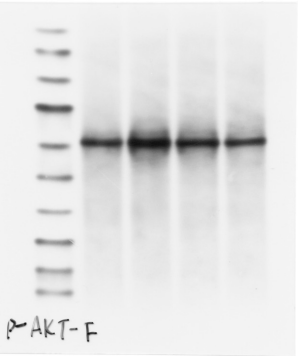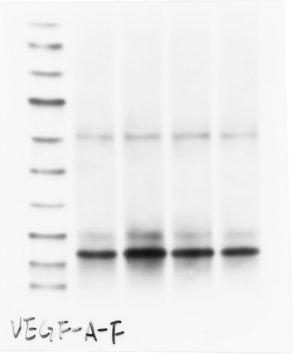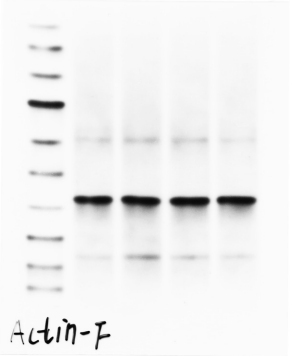

Repricate 2

|            |   |   |   |   |
|------------|---|---|---|---|
| PBS        | + | — | — | — |
| ADSCs-EVs  | — | + | + | + |
| CCN2 siRNA | — | — | + | — |
| LY294002   | — | — | — | + |

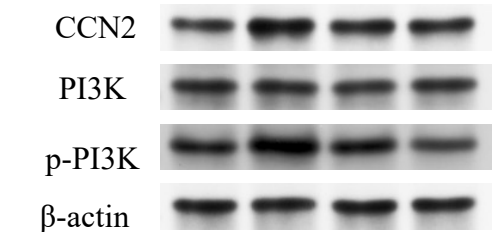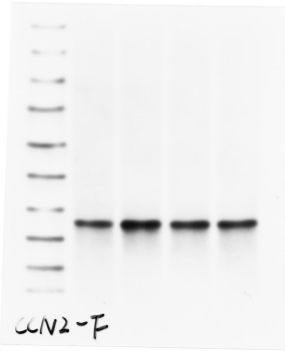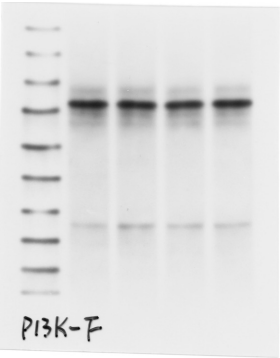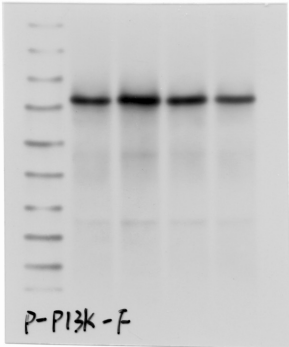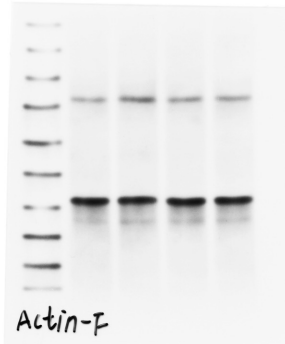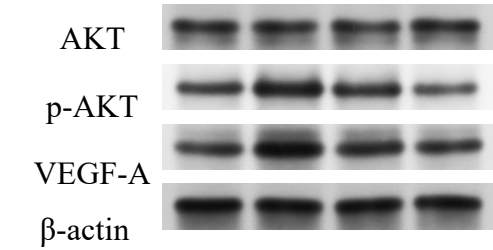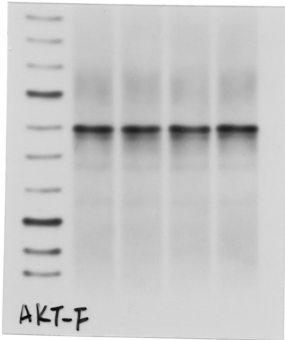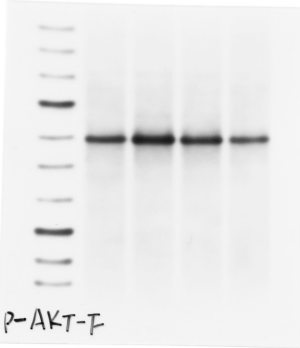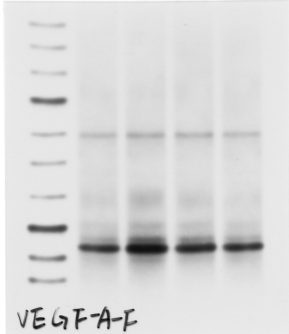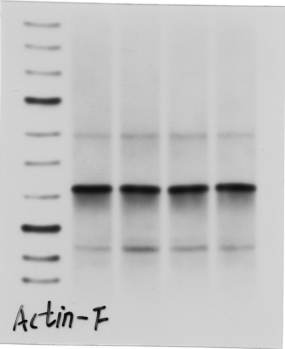

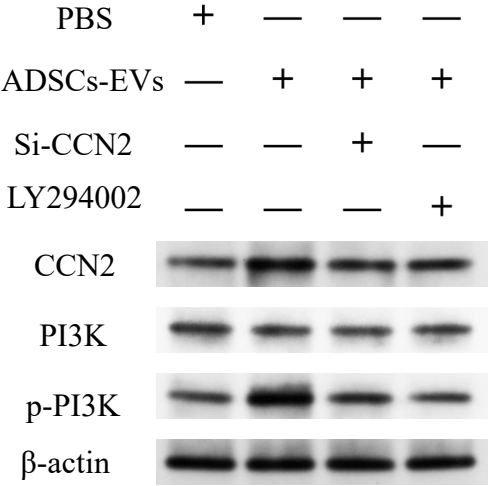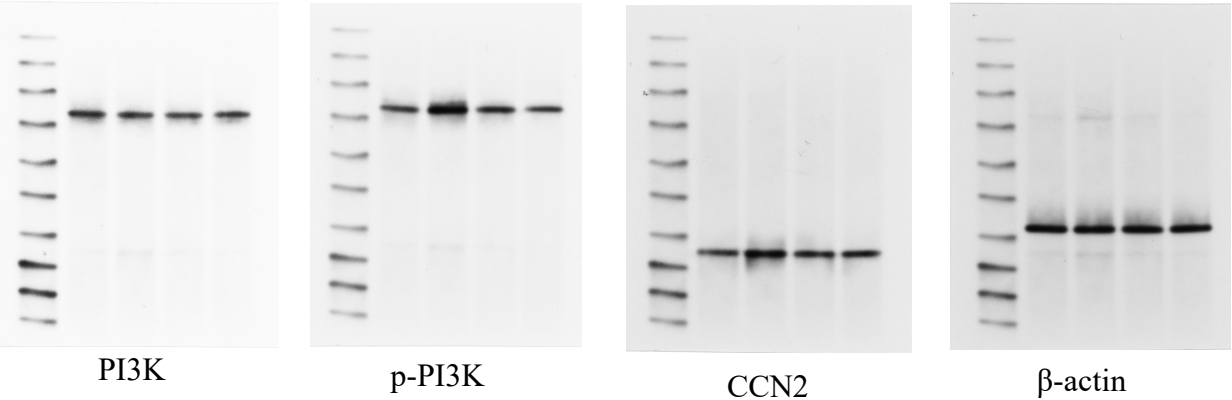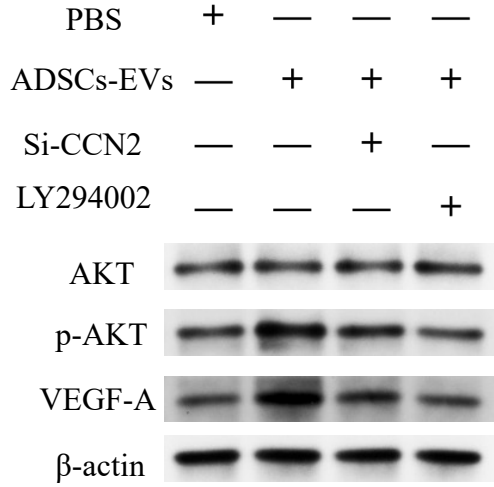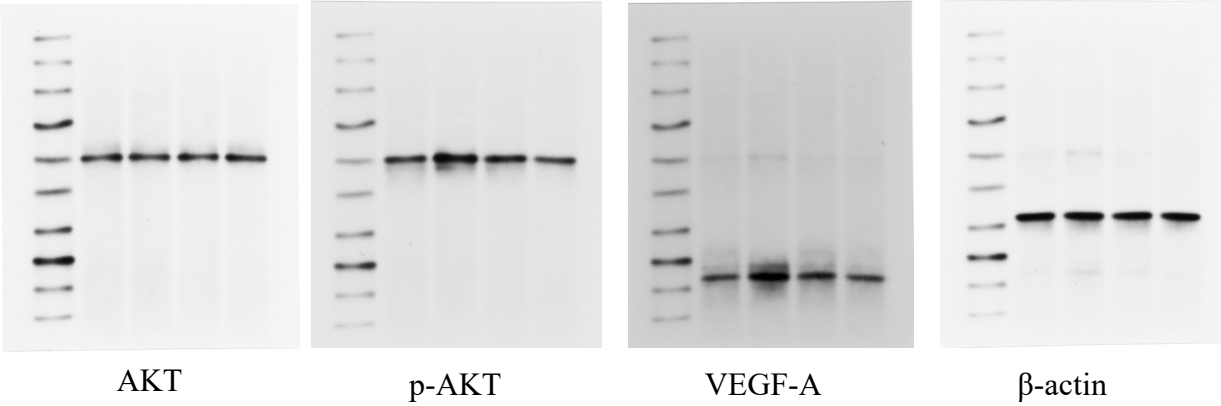

Supplement: Supplementary file 1 — Additional file 1. [file 13287_2025_4354_MOESM1_ESM.pdf]
